# Flexible corner cube retroreflector array for temperature and strain sensing†
†Electronic supplementary information (ESI) available. See DOI: 10.1039/c7ra13284k


**DOI:** 10.1039/c7ra13284k

**Published:** 2018-02-16

**Authors:** Muhammad Waqas Khalid, Rajib Ahmed, Ali K. Yetisen, Haider Butt

**Affiliations:** a Nanotechnology Laboratory, School of Engineering, University of Birmingham, Birmingham B15 2TT, UK. Email: h.butt@bham.ac.uk; Tel: +44 (0)1214158623; b School of Chemical Engineering, University of Birmingham, Birmingham B15 2TT, UK

## Abstract

Optical sensors for detecting temperature and strain play a crucial role in the analysis of environmental conditions and real-time remote sensing. However, the development of a single optical device that can sense temperature and strain simultaneously remains a challenge. Here, a flexible corner cube retroreflector (CCR) array based on passive dual optical sensing (temperature and strain) is demonstrated. A mechanical embossing process was utilised to replicate a three-dimensional (3D) CCR array in a soft flexible polymer film. The fabricated flexible CCR array samples were experimentally characterised through reflection measurements followed by computational modelling. As fabricated samples were illuminated with a monochromatic laser beam (635, 532, and 450 nm), a triangular shape reflection was obtained at the far-field. The fabricated flexible CCR array samples tuned retroreflected light based on external stimuli (temperature and strain as an applied force). For strain and temperature sensing, an applied force and temperature, in the form of weight suspension, and heat flow was applied to alter the replicated CCR surface structure, which in turn changed its optical response. Directional reflection from the heated flexible CCR array surface was also measured with tilt angle variation (max. up to 10°). Soft polymer CCRs may have potential in remote sensing applications, including measuring the temperature in space and in nuclear power stations.

## Abstract 2

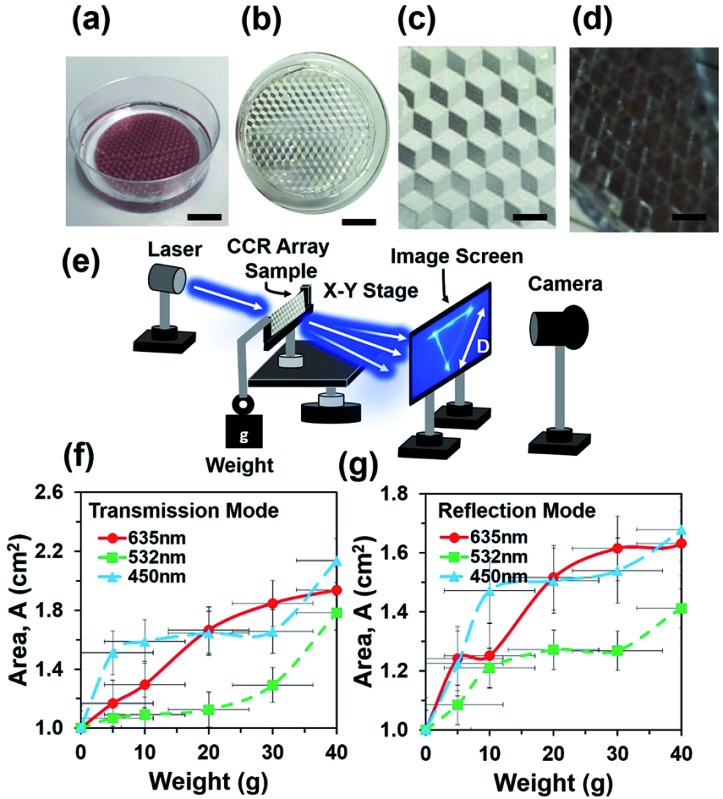
A flexible corner cube retroreflector (CCR) array based passive dual sensing is demonstrated to measure external stimuli (temperature/mechanical force as weight suspension).

## 1. Introduction

Soft, flexible optical sensors have been considered as an alternative to conventional planar, rigid and brittle electronic devices.^1^ Electronic sensors have active components and require a power supply to function, they have limitations such as high cost and manufacturing complexity, and they are prone to electromagnetic (EM) and thermal noise interference.^2^ Sensing platforms based on optical components to detect and monitor environmental factors such as humidity, pressure, shear, and torsion have applications in robotics, wearable devices, medical diagnostics, and healthcare monitoring.^3^–^8^ Flexible optical sensors due to the advantages of low cost, compactness, low noise/interference, high sensitivity and reliability have been utilized in temperature and strain quantification.^9^–^11^ However, these optical devices cannot sense temperature and strain simultaneously. Hence, the development of simple, cost-effective, and robust optical sensing technologies is highly desirable for remote sensing applications.

A corner cube retroreflector (CCR) is a reflector that consists of three mutually perpendicular intersecting flat mirror surfaces that produces unique retro-reflected light.^12^–^14^ Directional and optical phase conjugation (OPC) properties of a CCR array is based on reflection from the mirror surfaces.^15^,^16^ Directional property of a CCR array reflects light back to its source and is independent of illumination angle.^17^ Directional property of CCRs have applications in imaging, navigation, displays, sensors, optical communication, and low-powered sensor networks.^18^–^23^ The OPC of a CCR is an optical phenomenon in which an optical phase conjugated wave (OPW) is generated by reversing the phase of the incident electromagnetic wave at each and every points.^24^–^26^ The OPC property of CCRs have applications in optical interferometry, tomography, near-field microscopy, sensing, wavefront reconstruction and corrections.^15^,^16^,^19^,^25^,^27^ Although perfect CCRs are always desired to produce retroreflection light,^28^ imperfect CCRs also have practical applications in traffic signals or car lights.^21^,^29^ Imperfect CCR produces quasi-retroreflected light formed through intentionally introduced artifacts or structural imperfections during their fabrication process.^28^ CCR fabrication is based on a range of methods including microelectromechanical methods, nanoimprinting, lithography, and direct laser writing.^30^–^32^ CCRs have also been fabricated through mask-based direct etching methods, diamond micromachining, and laser ablation techniques.^31^,^33^,^34^ These fabrication techniques are limited as they are expensive and expertise-dependent, require advanced equipment, complex and time-consuming processes. Recently, fast and low-cost holography techniques have been used to fabricate CCRs and miniature diffractive optical devices (lens, diffusers, and gratings).^15^,^21^,^35^

Here, we demonstrate a method to rapidly produce optical sensors composed of a CCR array structured in polymer polydimethylsiloxane (PDMS), through an embossing process. This fabrication is robust, flexible, low cost, simple, immune to EM and thermal noise interference and is passive (*i.e.* no power supply needed). PDMS based sensors can be useful in harsh environments due to their stable chemical properties.^36^–^38^ Transmission of light of a desired wavelength could be achieved by an appropriate concentration of a doping dye, PDMS is a non-toxic and inert silicon-based organic polymer capable of replicating 3D structures in microscale^39^ and has been a popular choice for soft lithography due to its robust nature, low cost and ease of fabrication to replicate microscale structures.^33^ As compared to traditional etching and bonding approaches, PDMS microfabrication is rapid and simple. PDMS has a refractive index of ∼1.4 and is transparent in the visible range (400–800 nm),^40^ and inert properties make PDMS suitable for prototyping and testing.^41^

Optical properties of the fabricated flexible CCR array were characterized through reflection, transmission, far-field experiments as well as numerical modelling. For monochromatic light illumination of the flexible CCR array, a far-field triangle response was formed on the image screen. Any perturbation (expansion or compression), either due to mechanical stress or thermal effects, altered internal angle size of the fabricated flexible CCR array and therefore optical response (retroreflection or far-field triangular structure) changes accordingly. The retroreflected light from the flexible CCR array was tuned through temperate, applied force by weight suspension and inward/outward bending. The profile of light transmitted/reflected through/from an elastomer with the cornercube array structured on its surface depended on the degree of compression or expansion of flexible CCR array. In general, the retroreflected power decreased considerably from 10° onward with increased temperature.

## 2. Results and discussion

### 2.1 Sample preparation and CCRs replication

**Fig. 1:**
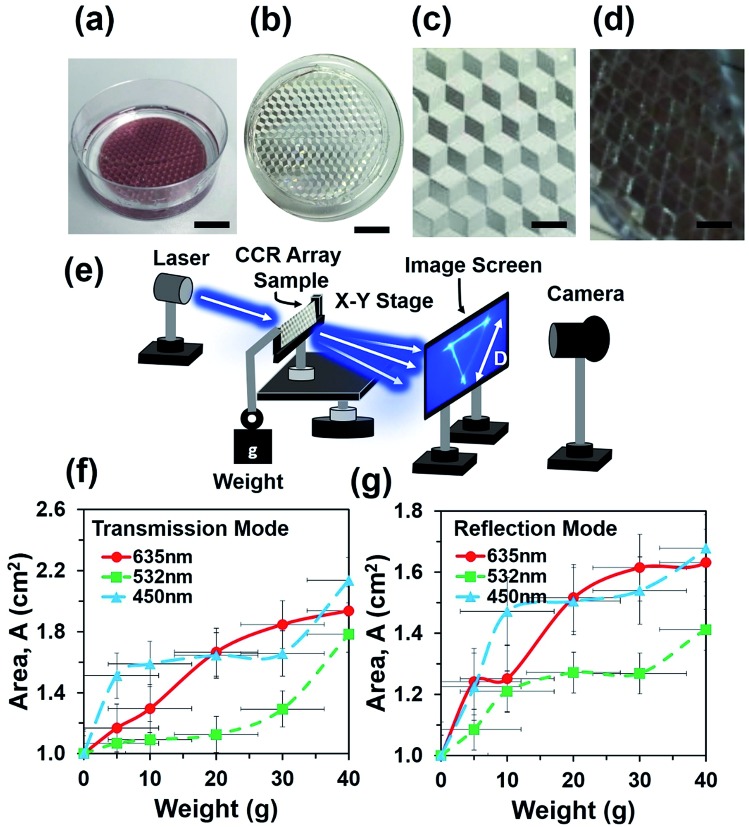
Schematic diagram (a) sample fabrication *via* PDMS replication method. Scale bar = 1.5 cm. (b–d) Replicated flexible CCR array, magnified version of without and with Au coated samples. Scale bars = 1.5, 0.2 cm. (e) Schematic experiment setup for optical characterization during transmission mode. (f and g) Reflection area of transmitted and reflected triangle as a function of weight suspension.

Flexible CCR arrays were fabricated using mechanical stamping/embossing process (Fig. S1a†). The PDMS polymer solution was prepared by mixing PDMS monomer and curing agent (10 : 1, v/v). The mixture was mixed for 20 min by a magnetic stirrer and followed by ultrasonic cleaning to remove air bubbles from the mixture. CCR arrays were placed in a Petri dish and fixed with rotary stage of a spinner. The PDMS precursor mixture was poured into the dish and rotated through 400, 600, and 800 rpm for uniform distribution on the top of CCR mold. Different levels of chemical mixtures were poured into the Petri dish and rotation speed was increased to make thicker to thinner samples (Fig. 1a). Samples were kept in at 50 °C for 3 h to cure the mixture. The fabricated PDMS replica was peeled off from the original CCR array mold. Replicated structures were immediately ready for optical sensing in transmission mode (Fig. 1b). The fabricated flexible CCR array consisted of internal three mirror surfaces (Fig. 1c). For further examinations of the CCR array and to check the feasibility in reflection mode, a 20 nm thick layer of gold coating was sputtered on the surface to increase the reflectivity. The fabricated coated samples consisted of microcubic-corner retroreflector (MCCR) array structures, where three mirror-reflection planes were in hexagonal patterns (Fig. 1d).

### 2.2 Optical properties, computational modelling and optical characterization

The optical property of flexible CCR arrays is based on the total internal reflection (TIR) reflection effect.^15^ Therefore, an incident light reflected three times from each mirror plane once and become a retroreflected beam. However, not all light entering to a cornercube reflects three times to become part of retroreflection.^21^ Light entering at the center of the cornercube (active zones) has more probability to be retroreflected than that of entering at the sides.^42^ The retroreflected light is phase conjugated to the incident light. Fig. S1b† shows optical reflection property from a plane mirror, a rigid CCR array, and flexible CCR array. All three optical devices change the direction of normal component of the reflected light, although a plane mirror does not change the amplitude and phase component of the reflected light (eqn (2)). As incident angle changes the reflection angle also changes, obeying Snell's law. However, the CCR array changes both amplitude and phase components of the reflected light (eqn (3)). The incident light retroreflected from the CCR array and is independent of illumination angle. The flexible CCR array changes internal angle and size of the three mirror surface due to external stimuli (temperature, humidity, stress and strain, *etc.*). Therefore, retroreflection property works up to certain illumination angle and light scatters at larger angles and is unable to fulfil three mirror based retroreflection (eqn (4), Fig. S2†). Directional reflection property of a flexible CCR array can be expressed based on wavefront analysis. For an arbitrary incident plane wave (eqn (1)) under paraxial approximation (*K*_*z*_ = *K* = 2π/*λ*):

1 *E*_i_(*x*,*y*,*z*) = *A*(*x*,*y*)e^(i*φ*(*x*,*y*)–i*K*_*z*_)^

The reflected light from the plane mirror is:

2 *E*_PM_(*x*,*y*,*z*) = *A*(*x*,*y*)e^(i*φ*(*x*,*y*)+i*K*_*z*_)^

The reflected light from CCR array is:3
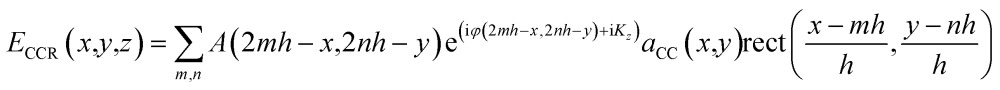



The reflected light from a flexible CCR array is:4
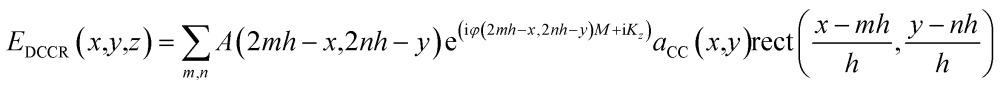
where *A*(*x*,*y*), *φ*(*x*,*y*) and rect(*x*,*y*) represent amplitude, phase and rectangular functions, respectively. *m* and *n* are optical field segments of the reflected light by each single CCR. *m* represents the optical phase modulation of reflection due to size change of the flexible CCR array. *a*_CC_(*x*,*y*) is a scalar quantity defined as the aperture function of the reflected beam amplitude. rect(*x*,*y*) represents a rectangular function and defined as 1, where abs(*x*,*y*) < 1/2 and 0 otherwise.^21^ Optical characterization of the flexible CCR was performed using monochromatic light illumination and a far-field experimental setup (Fig. 1e). Upon illumination with a monochromatic light source, the flexible CCR array produced a triangular shape on the image screen in both transmission as well as reflection mode. The spot size of the incident laser beam was larger than the dimensions of a single cornercube structure. The incident light transmitted through each plane once as well as some part of incident light was reflected and propagated along plane-to-plane of the corner cube, all these segments of light interfere and produced phase conjugated interference pattern in the form of a triangle at the far field. In transmission mode, a sample holder was used to keep the sample fixed and the sample was illuminated in the normal direction with monochromatic. In reflection mode, monochromatic light was illuminated at 30° tilt angle. The reflected/transmitted light produced a far-field triangular interference pattern on the image screen, which was captured using a digital camera. External weight was added at the end of sample holder to physically expand the elastomer CCR array sample. Fig. 1f and g show the strain sensing response of the flexible CCR as a function of the external load. During transmission/reflection mode, as the weight increased, the size of interference triangle increased. Reflected or transmitted light through/from the flexible CCR array depended on internal angle variation. Therefore, minimum or maximum reflection distance between two interference points of the triangular structure changed due to physical expansion or compression of the flexible CCR array structure. During reflection/transmission mode, minimum reflection distances were measured with lower weights and normal green (532 nm) light illumination.

**Fig. 2:**
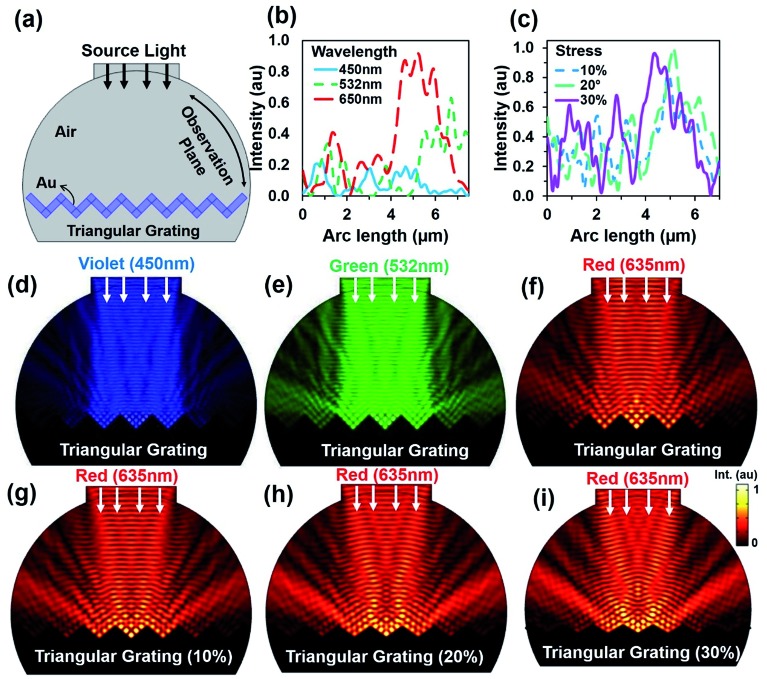
Computation modeling of light reflection from triangular grating structure with normal light illumination. (a) Schematic diagram of FEM simulation. (b) Reflected light intensity with monochromatic normal light illumination. (c) Reflected light intensity with stress variation. (d–i) Electric field distribution with monochromatic light illumination and stress variation.

As the CCR array expanded due to high strain or temperature variation, the angle between the CCR structures increased. Dynamic optical property was simulated through a computational model based on finite element method (FEM) meshed in COMSOL Multiphysics.^43^ Fig. 2a shows a hemispherical block diagram for FEM simulation of the flexible CCR array. The CCR array was considered as a triangular grating structure (side view). Therefore, compression or expansion of the flexible CCR array was considered as the variation of triangular grating structures. Fig. S3a and b† show a 3D simulation diagram and associated mesh diagram with the variation of triangular grating structures. Fig. 2b shows reflected light intensity as a function of arc length of incident laser wavelength. As the wavelength increased, the reflected light intensity also increased. Therefore, minimum and maximum light reflections were observed with violet (450 nm) and red (635 nm) light under normal illumination respectively. Further simulations were also performed with the expansion of corner cube structures and associated light reflection properties. Fig. 1c shows reflected light intensity as a function of arc length due to the triangular mesh geometry variation 10%, 20% and 30% from normal geometry (having 90° triangular angle). Fixed wavelength (635 nm) was considered during triangular structural variation due to strain. However, similar simulation results were also observed with violet (450 nm) and green (532 nm) light illumination with triangular structural variation (Fig. S4 and S5†). Fig. 1d–f shows electric field intensity distribution due to incident wavelength variation. Maximum and minimum light reflection from the triangular grating structures were observed due to red (635 nm) and violet (450 nm) light illumination. The retroreflection property was valid for any illumination wavelength. Similarly, Fig. 1g–i shows electric field intensity distribution due to triangular structure variation with fixed incident wavelength (635 nm). Maximum and minimum light reflection from the triangular structures were observed due to maximum (30%) and minimum (10%) from normal illumination. Similar light reflection field intensity distribution was also observed with green and violet light normal illumination (ESI, Fig. S4 and S5†). Maximum light reflected toward the source reduced as compared to a fixed CCR size. However, retroreflection property was also valid with CCR array size variation and at fixed illumination wavelength.

**Fig. 3:**
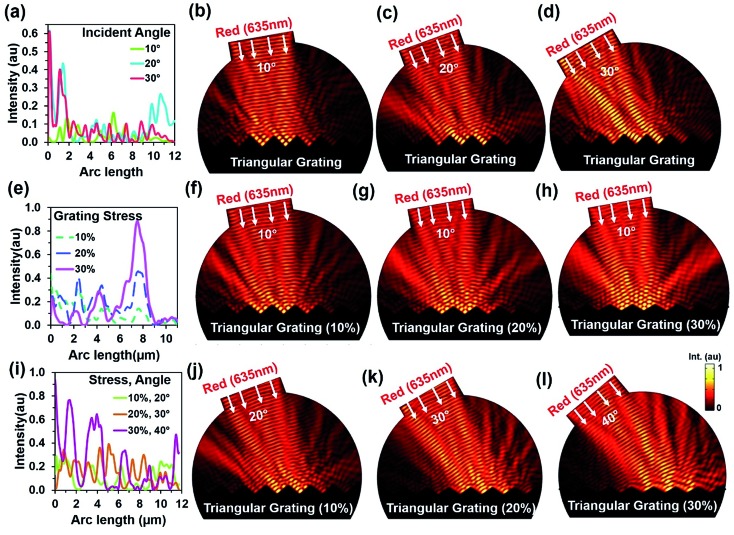
Computation modeling of light reflection from triangular grating structure with tilted light illumination. (a–d) Reflected light intensity and electric field distribution and with tilted illumination (10, 20, and 30°) and fixed triangular grating structure. (e–h) Reflected light intensity and electric field distribution and with fixed tilted illumination (10°) and triangular grating structure expansion (10, 20, and 30%) from normal. (i–l) Reflected light intensity and electric field distribution with tilted illumination (20, 30, and 40°) and triangular structure expansion (10, 20, and 30%) from normal.

Further simulations were performed to observe light retroreflection with fixed and triangular grating structural variation at tilted red (635 nm) light illumination. Fig. 3a shows reflected light intensity with illumination angle variation. Generally, low reflection was observed at lower tilted angles. However, light reflection had fewer influxes with triangular grating structure due to direction property of CCR structure. Fig. 3b–d show electric field intensity distribution with 10°, 20°, and 30° tilt illumination. Directional reflection intensity toward the source increased with tilted illumination. Fig. 3e–h show light retroreflection, reflection property with fixed tilt angle 10° and triangular structure 10%, 20% and 30% expansion from the normal. As the triangular structure expanded, the reflected light intensity also increased. Therefore, maximum (90%) and minimum (20%) light reflection was observed at 30% and 10% expansion of triangular structure from the normal. Fig. 3f–h show retroreflection, reflection electric field light distribution with (10% to 30%) and fixed angle (10°) illumination. Maximum light reflection field intensity distribution was observed at 30% triangular structure expansion. Fig. 3i–l shows light reflection property with tilt angle (20, 30, 40°) variation and triangular structure expansion (10%, 20% and 30%) from the normal. As tilt angle and triangular structure expansion increased, reflected light intensity also increased. Maximum (90%) and minimum (30%) light reflection from triangular structure were observed at maximum and minimum tilted angle and triangular structure expansion from the normal. Fig. 3i–l shows electric field intensity distribution with tilted illumination and triangular structure expansion. Maximum light reflection distribution were observed with maximum tilted angle (40°) and triangular structure expansion (30%) from the normal. Similarly, light reflection was also observed with triangular structure variation, green and violet light at tilt angle variation (ESI, Fig. S6–S9†). In all the electric field intensity distributions, maximum light reflected toward the source and showed retroreflection property and its validity with structural variation.

**Fig. 4:**
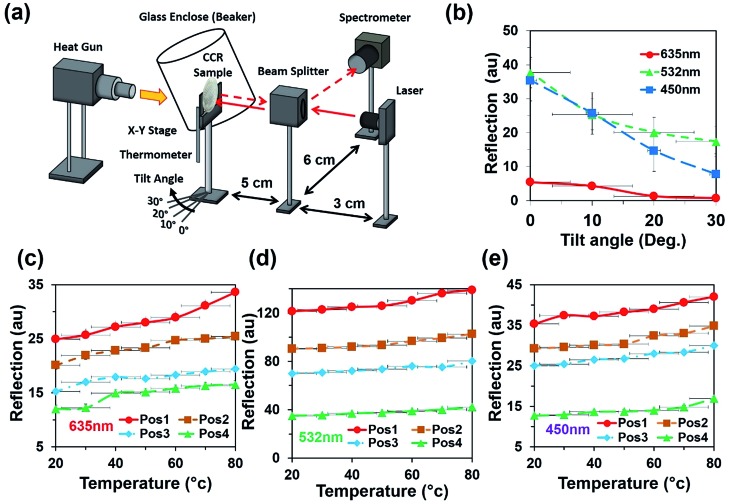
Directional reflection of flexible CCR array with temperature variation. (a) Schematic experiment diagram for selective directional reflection measurement with temperature and angle variation. (b) Directional reflection of flexible CCR array as a function of tilted angle variation with red, green, and violet light normal illumination. (c–e) Directional reflection of flexible CCR array as a function of temperature variation with red, green, and violet light normal illumination at four different positions (pos1, pos2, pos3, and pos4).

Light retroreflection from the triangular structure was predicted from the computational modeling. Based on computational results, optical experiments were performed to observe the flexible CCR's response with triangular structure variation. The retroreflection property of the fabricated flexible CCR structure was observed through reflection measurements. One of the important attributes of the CCR array is its directional property, *i.e.* incident light is reflected towards the source at any illumination angle. The fabricated flexible CCR structure strongly followed this directional property. To observe the directional property of the flexible CCR array and tune its optical property with external stimuli, retroreflective light was measured through an established optical setup (Fig. 4a). Light was illuminated from a laser source, passed through an input port of a polarization-independent beam splitter. Finally, reflected light intensity from the Au coated flexible CCR sample was measured through a spectrophotometer. To observe temperature-dependent expansion of the flexible CCR array structure and associated reflected light intensity variation, constant heat flux was supplied from a hot air blower (heat gun). A glass enclosure was used to ensure uniform temperature distribution and to reduce vibrational effects from the flow of heat. A mercury thermometer was used to measure the temperature of the enclosure. The experimental setup was placed on railings so that distance between the sample and beam splitter could be altered. The flexible CCR sample was held by an *x*–*y* positioning stage. This allowed the sample to be repositioned so the laser focus position could be chosen and was able to rotate at predefined angles to observe angle-dependent directional reflection. Heating the sample expanded the corner cube structures, which in turn enlarged the size of the reflected triangle and affected the optical response of CCR array. A direct relationship was observed between the temperature and reflected optical power for PDMS CCR arrays.

Sample without any weight suspension was illuminated with various light sources (635 nm, 532 nm, and 450 nm) and the optical response was recorded on a far-field screen (a triangle). In the next step, weight was suspended from the free side of sample and increased from 10 g to 40 g. Increasing the weight elongated the cornercube array *via* mechanical strain force. Each increment of weight increased the degree of expansion of each cornercube structure within the array, which in turn affected the size of the transmitted triangle on the far screen. A direct relationship between increasing load and magnitude of the transmitted power (area of transmitted/reflected triangle) was measured consistently. For each weight suspension, the optical response (triangle) was recorded on the far-field screen with a digital camera. Furthermore, the sample was coated with a gold layer and kept at an angle of 120° to the light source and far-field screen to obtain data in reflection mode. In general, a direct relationship between mechanical stresses towards optical response was found; for each 0.02 N increment of applied inward force, this increased the area of transmitted triangle up to 0.2 cm^2^. However, strain produced by a specific force depended upon the thickness of the fabricated PDMS block (sample).

Fig. 4b shows directional reflection intensity as a function of tilt angle. At room temperature, directional reflected light intensity was measured with normal red, green, and violet light illumination. Maximum and minimum light intensities were recorded with green and red lights illumination. Directional reflection intensity was also measured with temperature variation with red, green, and violet light illumination (Fig. 4c and d). As temperature increased, reflected light intensity also increased. Reflection was measured at four different positions. Maximum and minimum directional reflections were observed with green and red lights illumination respectively, with high and minimum temperature. Therefore, the directional reflection amount was tuned through temperature variation.

**Fig. 5:**
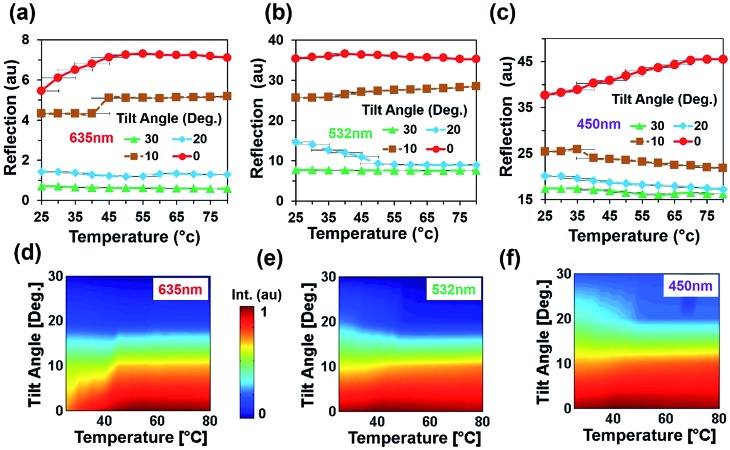
Directional reflection of flexible CCR array with temperature and tilt angle variation. (a–c) Directional reflection of flexible CCR array as a function of temperature and tilted angle variation with red, green, and violet light normal illumination. (d–f) Directional reflected light intensity as a function of temperature variation with red, green, and violet light normal illumination.

An angular directional reflection experiment was performed to measure the optical response of the flexible CCR array with temperature and tilt angle variation. Fig. 5a–c shows direction reflection from the flexible CCR with temperature (25–75 °C) and tilt angle (0–30°) variation with red (635 nm), green (532 nm) and violet (450 nm) light illumination. Maximum directional reflection was found at normal illumination (0°) and minimum reflection was found at maximum tilt angle (30°). Moreover, green and red light reflected maximum and minimum amount of light. Fig. 5d–f shows reflected light intensity as a function of temperature. For red, green, and violet light illumination, maximum intensity was at smaller tilt angle (<10°).

**Fig. 6:**
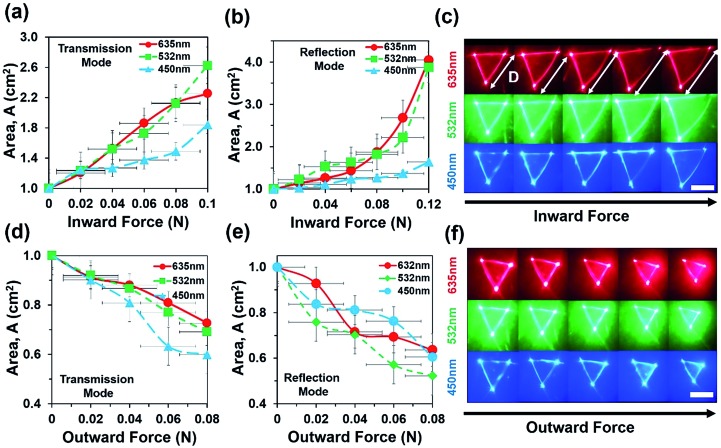
Force sensing of flexible CCR array with inward and outward bending during transmission and reflection mode. (a and b) Reflection area of far-field reflection triangle as a function of inward force with red, green, and violet light illumination. (c) Far-field reflection triangle due to increased inward force. Scale bar = 0.5 cm. (d and e) Reflection area of far-field reflection triangle as a function of inward force with red, green, and violet light illumination. (f) Far-field reflection triangle due to increased inward force. Scale bar = 0.5 cm.

Optical experiments were also performed to measure optical response of the flexible CCR array due to inward and outward bending force in transmission and reflection modes (Fig. 6). Optical response of the sample toward applied force (compression or expansion) was captured from the image screen *i.e.* triangular profile, which stayed the same until threshold value for perturbation was reached. Above this threshold force, optical response suddenly increased. The intensity *I*, of a laser at a point was defined as the energy per second per unit of area arriving at that point normal to the propagation direction (*I* = power/area = *P*/*A*). For a triangle with the base length *b* and height *h*, intensity can be expressed as *I* = 2*P*/*bh*. Change in the transmitted/reflected light intensity, *I*_c_ can be empirically correlated with the strain as *I*_c_(*ε*) = *I*_0_, if *ε* < threshold or otherwise, *I*_c_(*ε*) = *ε* × *I*_0_ – threshold, where *ε* is strain (degree of change in expansion due to applied force divided by initial structure without any force application), *I*_c_ is the change in intensity after force application and *I*_0_ is the initial intensity of light when no force is applied.

Fig. 6a and b shows area of reflection triangle as a function of inward force during transmission and reflection modes. As inward force increased, the area of transmitted/reflected triangle increased with red, green and violet light illumination in reflection and transmission modes. Fig. 6b depicts area of reflected triangle increased up to 0.9 cm (90%) as a result of 0.08 N applied inward force. During inward bending, the internal CCR structure and associated angle of planes reduced. Therefore the far-field reflected light produced a larger reflection triangle with increased inward force.^21^ Fig. 6c shows the far-field reflection triangle with increased inward force and was captured through an image screen setup. Similarly, Fig. 6d and e shows the area of the reflection triangle as a function of outward force during transmission and reflection modes. As outward force increased, the area of reflected profile decreased with red, green and violet light illumination in reflection and transmission modes. During outward bending, the internal CCR structure and associated angle of planes increased. Therefore, the far-field reflected light produced smaller reflection triangle as outward force increased. Fig. 6f shows far-field reflection triangle with increased outward force. Area of reflected triangle decreased up to 50% of the original area (*i.e.* no stress) due to 0.08 N of outward applied force.

The directional retroflection response of the flexible CCR array with internal triangular structure variation due external stimuli can be used as a temperature and strain sensor. Retroreflected light amount changed with temperature and strain variation. The reflected far-field triangular structure also increased/decreased based on temperature and strain variation. Optical response of the flexible CCR array worked in both reflection and transmission modes. Moreover, the amount of retroreflected light also depended on tilt angle. Therefore, reflected retroreflected light or triangular reflection area can be considered as a function of temperature and strain variation:5
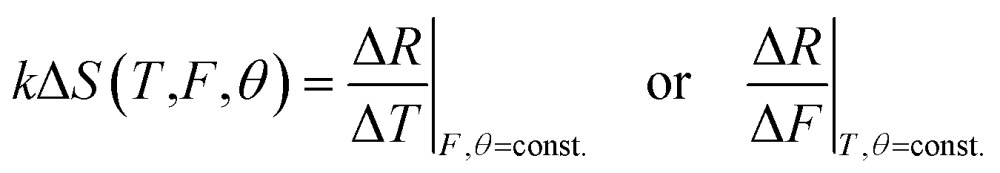
where *k* is a proportional constant and related with environmental conditions (relative humidity, temperature), Δ*R*, Δ*T*, and Δ*F* are changes in retroreflection, temperature, and force related to strain. Δ*S*(*T*,*F*,*θ*) is the change of sensitivity as a function of temperature, force related with strain or weight suspension and tilt angle of the flexible CCR sample. Therefore, sensitivity, *S* can be measured as a ratio of changes in retroreflected light or distance of far-field triangular pattern (d) and small change of temperature or strain variation (in the form of inward/outward force or weight suspension) during reflection or transmission mode (eqn (5)). At fixed illumination and without any load suspension or strain force, temperature sensitivity can be measured as *S*_T_ = 0.265 AU °C^–1^ (from the tangent of temperature response curve, Fig. 4d). Similarly, at fixed illumination and room temperature, strain sensitivity can be measured as *S*_S_ = 31.1267 cm^2^ N^–1^ (from the tangent of inward force, red (635 nm) illumination for response curve, Fig. 6b).

### 2.3 Discussion

The soft flexible CCR array showed TIR based on three mirror retroreflection. The incident light reflected toward the source with different tilt illumination and showed directional reflection. Flexible behaviour of the fabricated CCR array structure was observed with external stimuli (strain and temperature variation). In general, the directional reflection of the flexible CCR array was also observed with temperature and strain variation. The amount of retroflection varied with temperature, strain variation and provided selective directional reflection. As the CCR structures changed due to temperature or strain variation, the angle in the CCR structure increased/decreased, thickness of PDMS block changed which increased/decreased the size of the reflected/transmitted triangle and also changed the magnitude of retroreflection intensity. A direct relationship between temperature, strain, and retroreflected optical power from flexible CCR arrays was observed. In general, the reflected power of PDMS CCR increased with increasing temperature for all selected positions and monochromatic light illumination.

A gradual decrease in directional retroreflection occurred when the sample was tilted at larger angles. At normal angle (0° illumination), maximum reflection power was detected as all retroreflections were directed to the source which was redirected by the beam splitter to the optical spectrometer. As the tilt angle increased, some part of incident light might not reach inside the corner cube based on three mirror triangular meshes, scattered out by bulk PDMS block and redirected away from the beam splitter and hence by the optical powermeter, and retroreflection was not detected. Moreover, cross-sectional area of the corner cube array towards the incident ray may directly influence the retroreflected beam. Highest cross-sectional area was provided without tilting the sample resulted into highest retroreflection. Laser illumination on different sample positions resulted in different amount of retroreflection. Therefore the choice for first illumination point in each experiment was the position where the maximum reflection was found. The distance between the flexible CCR array, beam splitter, spectrometer, and laser source did not affect the retroreflection, given the focus point was fixed at a constant position. The detection limit (DOL), lowest detectable signals (temperature/force as weight suspension) can be calculated from the intercept between the regression lines of the standard errors. For red (635 nm) light illumination, the DOL values for temperature and weight variation are approximated at 30 °C and 5 g, respectively. However, DOL values are influence by incident light wavelengths, materials, and structural properties of the replicated CCR structure and metal-coating thickness.

For temperature or strain sensing, aperture size was an important factor which had to be positioned in a way that all the retroreflected light from the flexible CCR array reached the spectrometer. Light power will be only detected by the photometer if it passes through the aperture and reaches the detector. With increasing temperature, the flexible CCR array expanded, magnitude of reflected triangular profile became bigger than the size of aperture which may result into decreased power detected by the photometer (some light may scatter away in the surroundings) as detection power of the photometer was limited to the aperture size caped on it. *i.e.* In general, the detected power of PDMS CCR array due to thermal expansion increased with increasing temperature. During sensor sensitivity measurement, constant tilt angle (normal illumination) were considered for measurement simplicity. Moreover, external stimuli (humidity, temperature, strain force, and tilted illumination) may change the flexible CCR response in a complex way and reduce sensor's optical response and sensitivity. The directional reflection intensity and sensitivity of the proposed PDMS based CCR array sensor is low due to non-uniform gold coating. However the reflection intensity and sensitivity can be improved/enhanced by uniform selective coating as well as controlling the thickness of both PDMS replica and metal-coating.

## 3. Conclusion

We have successfully demonstrated directional retroreflection of a soft PDMS based flexible CCR array. The retroreflection property of a flexible CCR array was tuned through external stimuli (temperature and strain due to inward/outward force from weight suspension). Compared to a conventional CCR array, selective directional reflection was achieved using a flexible CCR array. Moreover, conventional CCR arrays are limited due to fixed retroreflection, but flexible CCRs allow tuning of retroreflection and are passive (no electronics required). Moreover, flexible CCR array based temperature and strain sensors described in this work were low cost, flexible and easy to fabricate. The sensitivity of a polymer based, flexible CCR array sensor could be customized with other copolymers or the PDMS CCR array could be coated with silver or gold nanoparticles to tune its optical–mechanical properties. Reflected optical power was independent of positioning and movement of the laser source. The direct relationship between force and magnitude of transmitted/reflected triangle was demonstrated in the flexible CCR array as a strain sensor. In addition, temperature and reflected power optical values were in agreement to prove the flexible CCR array could act as a temperature sensor. Sensors based on soft, flexible CCR arrays may have application in remote sensing as strain and temperature sensors. Applications for these optical sensors are in space science, where light waves can travel without being lost as heat to enable astronauts in space to measure the temperature and any deformation of their devices and parts of the spacecrafts by having a flexible CCR array installed on the surface, and directing a laser at it. Another application may be in nuclear powerstations and nuclear-related research where human operators measure temperature or nuclear expansion at a safe distances to ensure their safety and well-being from radiation and other environmental hazards.

## 4. Materials and methods

### 4.1 Materials and equipment

A CCR array was purchased from the JunAN (SL150-18, China) and used as a mold during embossing processing. Silicone elastomer base and curing agent (SYLGARD 184, 1.1KG) chemicals were purchased from Farnell, UK and used as a soft polymer embossing medium. Automatic sputter coater was purchased from the Agar Scientific, UK to make thin Au coating over flexible CCR array. COMSOL Multiphysics 5.2, MATLAB (Math Works, R2013) was used for the numerical simulations and data processing.

### 4.2 FEM modelling

COMSOL Multiphysics software based on FEM was used to model the flexible CCR array. Optical retroreflection/directional properties from the flexible CCR array were modelled through broadband light illuminated to the Au material based triangular grating surface at normal, and tilted angles (10, 20, 30, 40°). Temperature and strain effect on the flexible CCR array were simulated through 10, 20, 30% expansion of rectangular structure from normal. The reflected light from the triangular grating was measured from the hemispherical surface surrounded with air medium. The continuity and scattering boundary conditions were considered at triangular grating and hemispherical surface during FEM simulation. Sub-meshing (one fourth of incident light) and mesh convergence test was performed during simulation for the result accuracy. Triangular meshing elements was considered at the simulation domain. The maximum degree of freedom used was about 137 870. The completed mesh consisted of 701 boundary elements and 19 605 domain elements. The solution time was ∼30 s and ∼30 min during two and three-dimension (2D and 3D) simulation. Therefore (2D) simulation was performed to reduce additional computational complexity and time.

### 4.3 Flexible CCR array fabrication

Flexible CCR array fabrication was based on embossing process. Silicone elastomer base diluted with curing agent (10 : 1, v/v), mixed with magnetic stirrer in an ultrasonic bath to remove air bubbles. CCR array mold kept on a Petri dish and chemical mixture is poured into the dish and rotted through 400, 600, and 800 rpm through a spin coater (CHEMAT Technology, KW 4A) for thicker and thinner samples and uniform distribution of chemical on the top of CCR mold. Samples kept into the electric oven at 50 °C for 3–4 hours and dried samples remove from mold and sample ready for transmission mode.

### 4.4 Optical characterization

An optical spectrophotometer (resolution of ∼0.1–100 nm FWHM) was purchased from Ocean Optics for optical measurements. A C-Mounted Standard Cube Beamsplitters (38.0 × 38.0 × 50.0 mm^3^) was purchased from Edmund Optics, UK. Optical spectrophotometer, unpolarised beamsplitters, the fabricated flexible CCR array, and monochromatic light sources were used to optically characterise retroreflection property (Fig. 4a). The monochromatic light sources were used during transmission and reflection measurements through normal and tilted illumination at the flexible CCR array surface. The monochromatic light sources: red (635 nm, 4.5 mW, *Ø* 11 mm), green (532 nm, 4.5 mW, *Ø* 11 mm), and violet (405 nm, 2.6 mW, *Ø* 11 mm) were purchased from Thorlabs Elliptec GmbH (Dortmund, Germany). During transmission mode with PDMS CCR samples, reflection was not required, so samples were directly peeled off from the original CCR structure and used without any coating. However, a thin layer of gold coating with a thickness of 20 nm on the surface sample was applied to examine the feasibility of our samples operating in reflection mode. The PDMS sample has two surfaces that can be potentially coated with the gold coating. These are on the flat surface opposite the CCR structures, and the directly on the CCR structures. Second choice could affect the structures, so all tested PDMS CCR were gold-coated on the flat surface of the sample.

## Authors contribution

M. W. K. and R. A. fabricated samples, carried out optical experiments, simulations, analyzed results and wrote the article. A. K. Y. edited the article and made intellectual contributions. H. B. supervised simulation, experiments and led the project.

## Conflicts of interest

The authors declare no competing financial interest.

## Supplementary Material

Supplementary information

## References

[cit1] Do T. N., Visell Y. (2017). Sci. Rep..

[cit2] Ahuja D., Parande D. (2012). J. Sci. Res. Rev..

[cit3] Yeo J. C., Lim C. T. (2016). Microsyst. Nanoeng..

[cit4] Morin S. A., Shepherd R. F., Kwok S. W., Stokes A. A., Nemiroski A., Whitesides G. M. (2012). Science.

[cit5] Xu S., Zhang Y., Jia L., Mathewson K. E., Jang K.-I., Kim J., Fu H., Huang X., Chava P., Wang R. (2014). Science.

[cit6] Gong S., Schwalb W., Wang Y., Chen Y., Tang Y., Si J., Shirinzadeh B., Cheng W. (2014). Nat. Commun..

[cit7] Kaltenbrunner M., Sekitani T., Reeder J., Yokota T., Kuribara K., Tokuhara T., Drack M., Schwödiauer R., Graz I., Bauer-Gogonea S. (2013). Nature.

[cit8] Araci I. E., Su B., Quake S. R., Mandel Y. (2014). Nat. Med..

[cit9] Ding W., Jiang Y., Gao R., Liu Y. (2015). Rev. Sci. Instrum..

[cit10] Liu S., Yang K., Wang Y., Qu J., Liao C., He J., Li Z., Yin G., Sun B., Zhou J. (2015). Sci. Rep..

[cit11] Liu Y., Wang D., Chen W. (2016). Sci. Rep..

[cit12] Hansen J. P., Madhu S. (1972). Appl. Opt..

[cit13] Park B., Eom T., Chung M. (1996). Appl. Opt..

[cit14] Khalid M. W., Ahmed R., Yetisen A. K., AlQattan B., Butt H. (2017). Sci. Rep..

[cit15] Ahmed R., Yetisen A. K., Yun S. H., Butt H. (2017). Light: Sci. Appl..

[cit16] Chipman R. A., Shamir J., Caulfield H. J., Zhou Q.-B. (1988). Appl. Opt..

[cit17] Arbabi A., Arbabi E., Horie Y., Kamali S. M., Faraon A. (2017). Nat. Photonics.

[cit18] Bozhevolnyi S. I., Bozhevolnaya E. A., Berntsen S. (1995). J. Opt. Soc. Am. A.

[cit19] Baharav Y., Spektor B., Shamir J., Crowe D. G., Rhodes W., Stroud R. (1995). Appl. Opt..

[cit20] Zhou L., Kahn J. M., Pister K. S. (2003). J. Microelectromech. Syst..

[cit21] Ahmed R., Rifat A. A., Hassan M. U., Yetisen A. K., Butt H. (2017). RSC Adv..

[cit22] Wang S., Sherlock T., Salazar B., Sudheendran N., Manapuram R. K., Kourentzi K., Ruchhoeft P., Willson R. C., Larin K. V. (2013). IEEE Sens. J..

[cit23] Bergen M. H., Nichols J., Collier C. M., Jin X., Raja B., Roberts D. J., Ruchhoeft P., Willson R. C., Holzman J. F. (2014). Appl. Opt..

[cit24] Khalid M., Ahmed R., Yetisen A., AlQattan B., Butt H. (2017). Sci. Rep..

[cit25] Yaqoob Z., Psaltis D., Feld M. S., Yang C. (2008). Nat. Photonics.

[cit26] Shkunov V. V., Zel'Dovich B. Y. (1985). Sci. Am..

[cit27] Fainman Y., Lenz E., Shamir J. (1981). Appl. Opt..

[cit28] Park S.-W., Moon E., Chung H., Lee J.-Y., Bae C., Kim J.-W., Kim H. (2014). Appl. Opt..

[cit29] Kim H., Min S.-W., Lee B. (2008). Appl. Opt..

[cit30] Shaar N. S., Barbastathis G., Livermore C. (2015). J. Microelectromech. Syst..

[cit31] Lou Y., Wang H., Liu Q., Shi Y., He S. (2010). Appl. Opt..

[cit32] Sherlock T., Nasrullah A., Litvinov J., Cacao E., Knoop J., Kemper S., Kourentzi K., Kar A., Ruchhoeft P., Willson R. (2011). J. Vac. Sci. Technol., B: Nanotechnol. Microelectron.: Mater., Process., Meas., Phenom..

[cit33] Yuan J., Chang S., Li S., Zhang Y. (2002). Opt. Commun..

[cit34] Jolic K., Ghantasala M., Harvey E. C. (2003). J. Micromech. Microeng..

[cit35] Ahmed R., Yetisen A. K., El Khoury A., Butt H. (2017). Nanoscale.

[cit36] Lee L.-H., in Adhesives, Sealants, and Coatings for Space and Harsh Environments, Springer, 1988, pp. 5–29.

[cit37] Chung G.-S. (2007). Microelectron. J..

[cit38] Kim K. N., Chun J., Kim J. W., Lee K. Y., Park J.-U., Kim S.-W., Wang Z. L., Baik J. M. (2015). ACS Nano.

[cit39] Hassanin H., Mohammadkhani A., Jiang K. (2012). Lab Chip.

[cit40] Hoshino K., Shimoyama I. (2002). J. Micromech. Microeng..

[cit41] Jo B.-H., Van Lerberghe L. M., Motsegood K. M., Beebe D. J. (2000). J. Microelectromech. Syst..

[cit42] Yariv A. (1978). IEEE J. Quantum Electron..

[cit43] Ahmed R., Rifat A. A., Yetisen A. K., Dai Q., Yun S. H., Butt H. (2016). J. Appl. Phys..

