# Supplementary material for: Flexible corner cube retroreflector array for temperature and strain sensing
Source: RSC Adv. 2018 Feb 16;8(14):7588–98. doi: 10.1039/c7ra13284k (PMC5819368; doi:10.1039/c7ra13284k)
Supplement: RA-008-C7RA13284K-s001 [file RA-008-C7RA13284K-s001.pdf]

Electronic Supplementary Material (ESI) for RSC Advances.
This journal is © The Royal Society of Chemistry 2018

Supplementary Information

Flexible Corner Cube Retroreflector Array for Temperature and Strain Sensing

Muhammad Waqas Khalid,[1,] Rajib Ahmed,[1,] Ali K. Yetisen,[2] and Haider Butt[1,]*

[1]Nanotechnology Laboratory, School of Engineering, University of Birmingham, Birmingham B15 2TT, UK.

[2]Harvard-MIT Division of Health Sciences and Technology, Harvard University and Massachusetts Institute of Technology, Cambridge, MA, 02139, USA.

*E-mail: h.butt@bham.ac.uk, Tel.: +441214158623

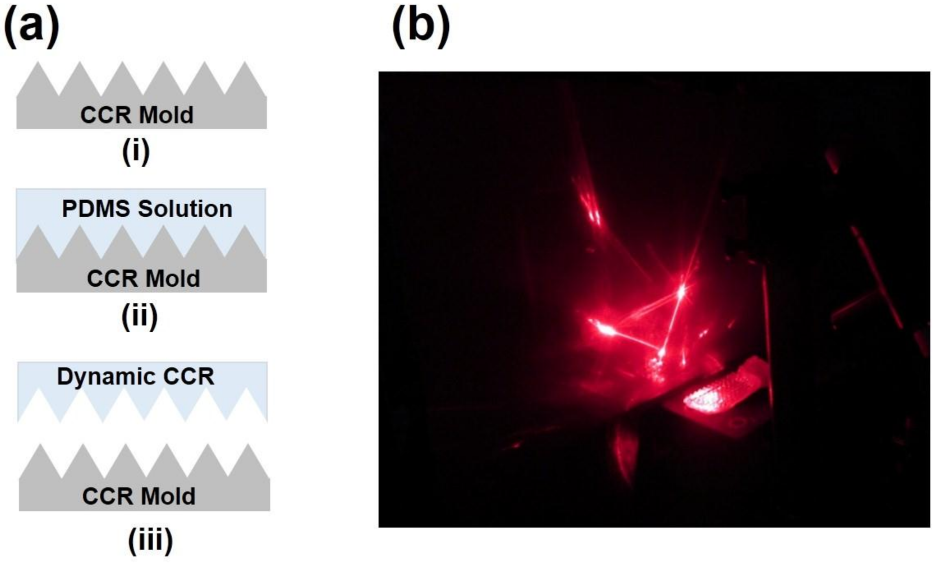

Figure S1: (a) Dynamic CCR fabrication (b) optical characterization through reflection mode.

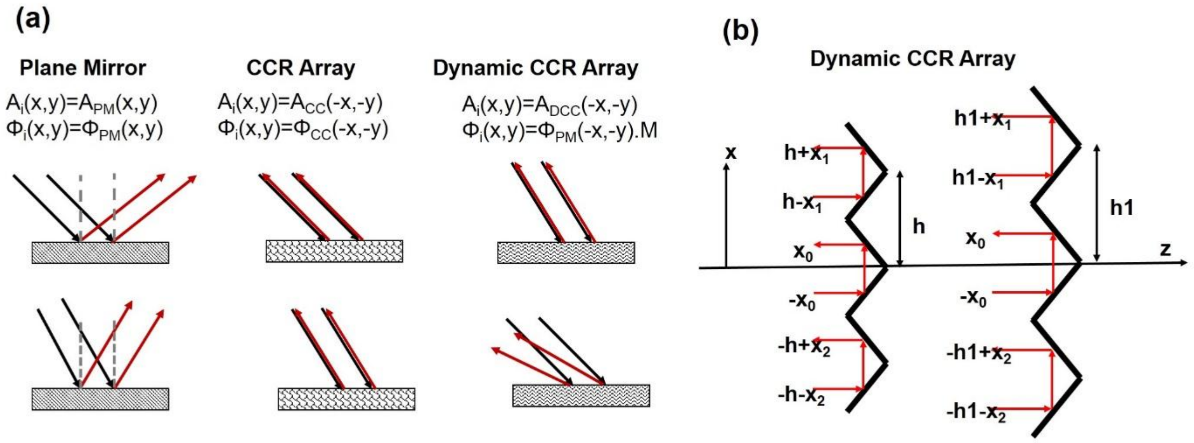

Figure S2: (a-b) Reflection and retroreflection property of plane mirror, CCR array, and dynamic CCR array.

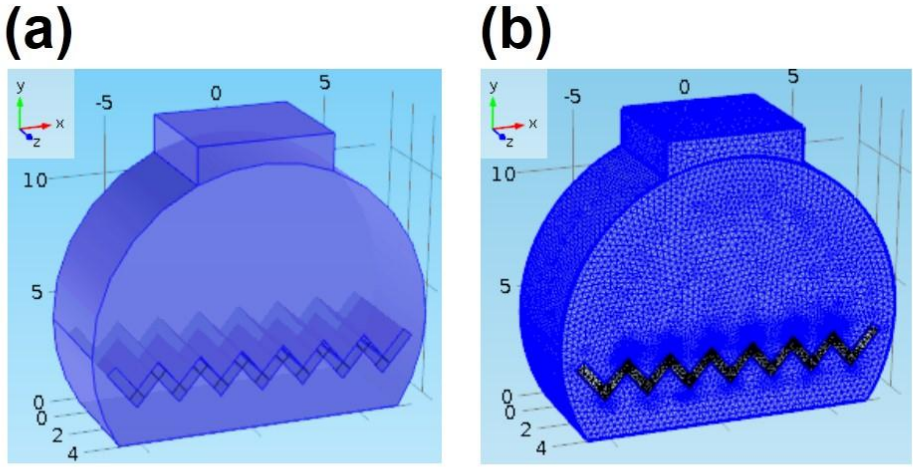

Figure S3: (a-b) 3D simulation and mesh diagram.

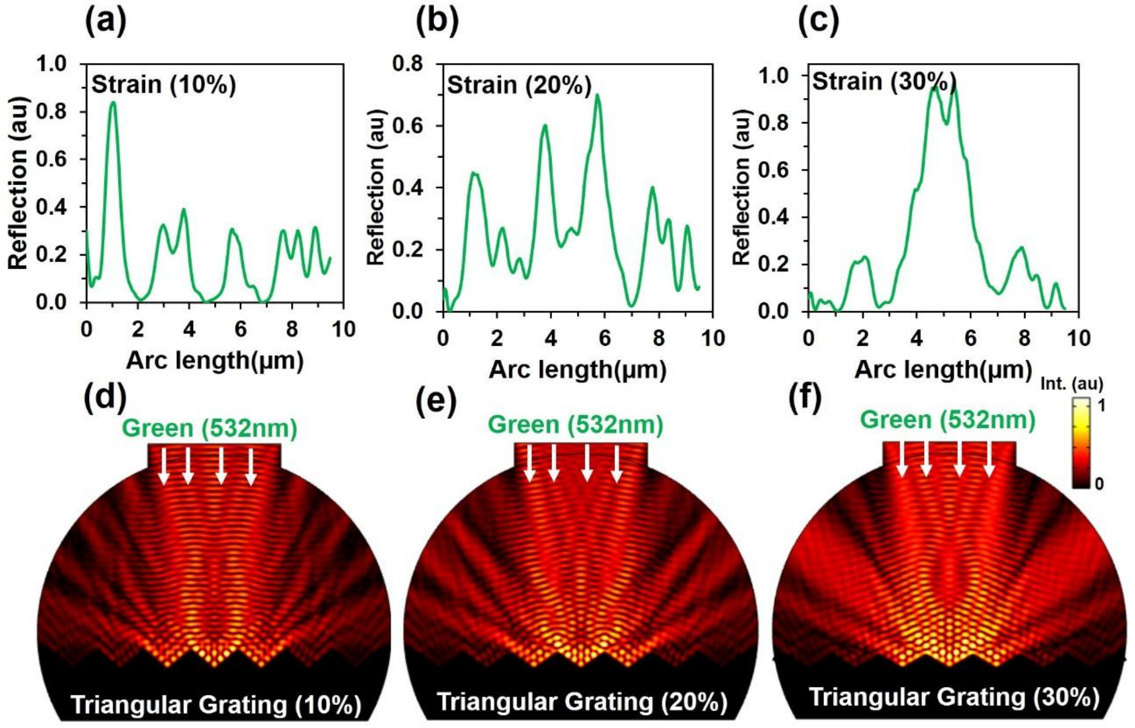

Figure S4: Computation modeling of retoreflection, diffraction/scattering from triangular grating structure with strain variation (10, 20, 30%) and green light illumination.

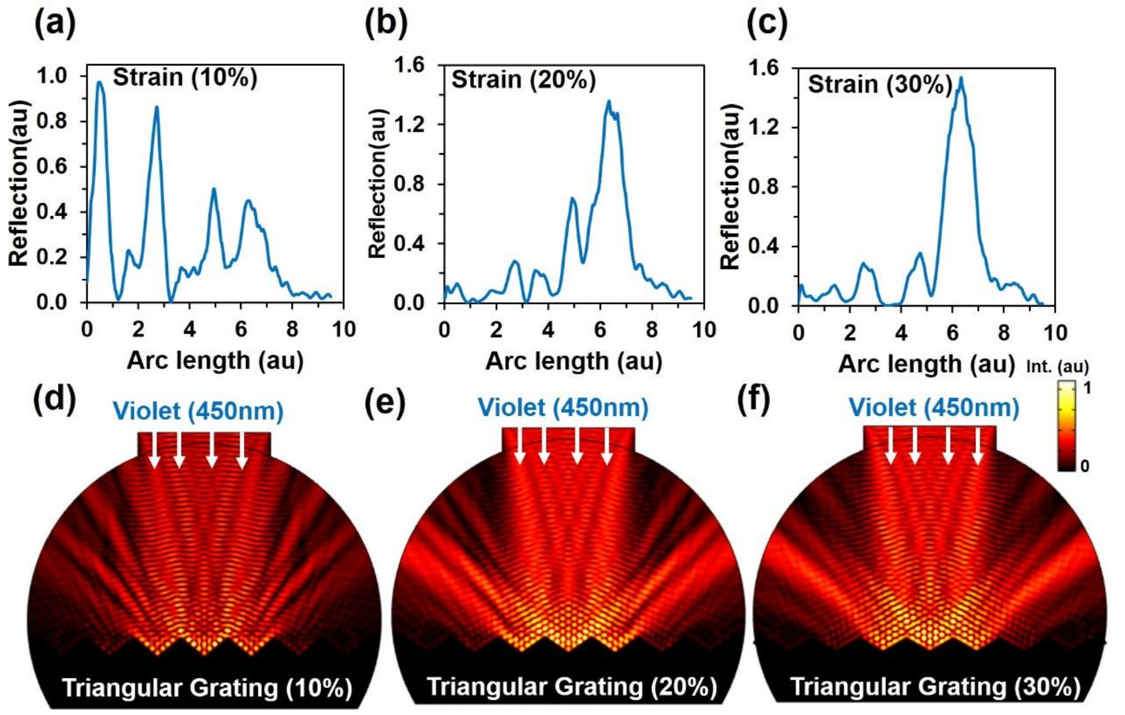

Figure S5: Computation modeling of retoreflection, diffraction/scattering from triangular grating structure with strain variation (10, 20, 30%) and violet light normal illumination.

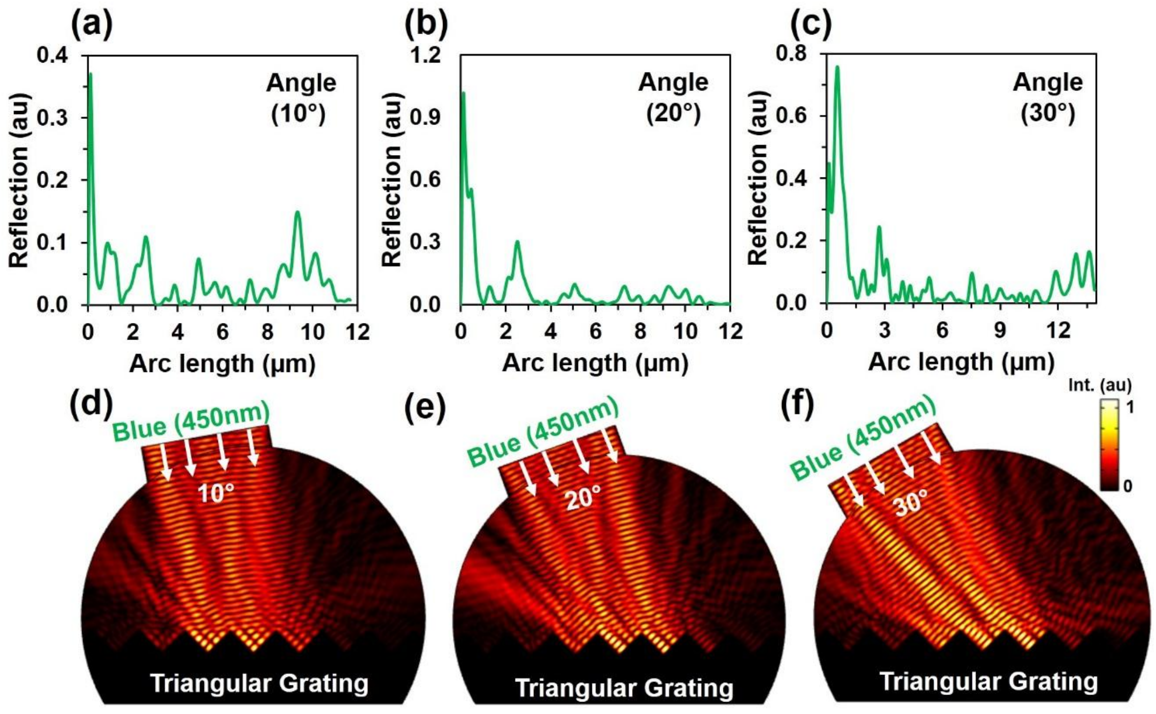

Figure S6: Computation modeling of retoreflection, diffraction/scattering from triangular grating structure with green light illumination angle variation.

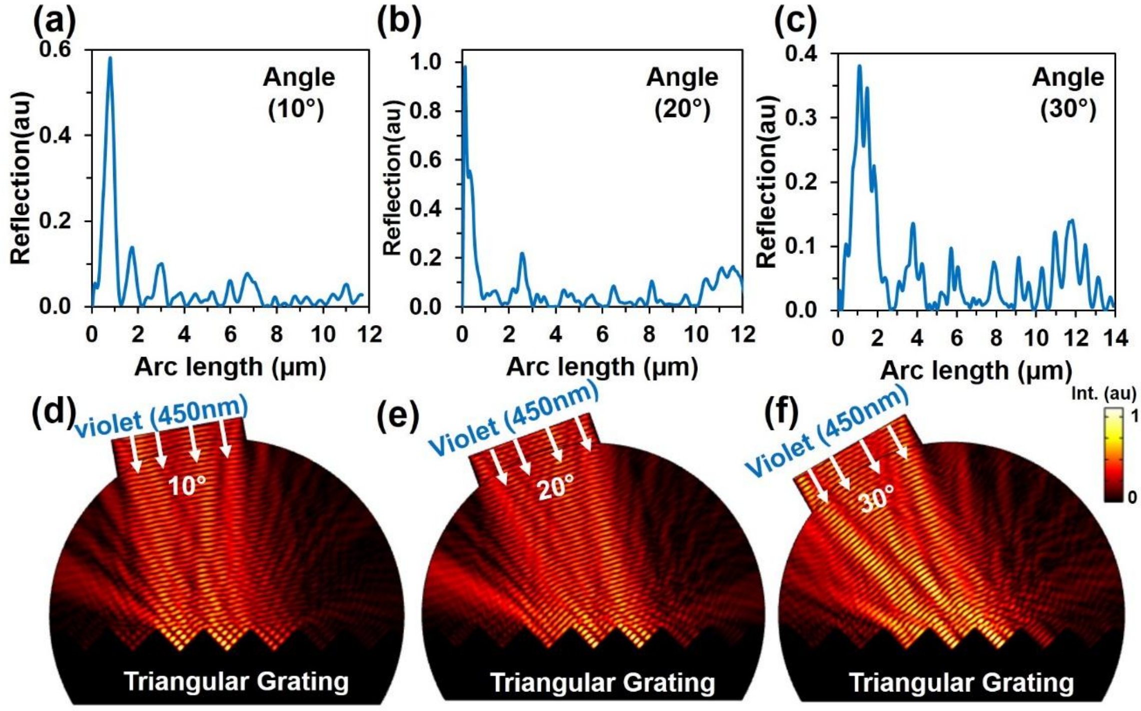

Figure S7: Computation modeling of retoreflection, diffraction/scattering from triangular grating structure with violet light illumination angle variation.

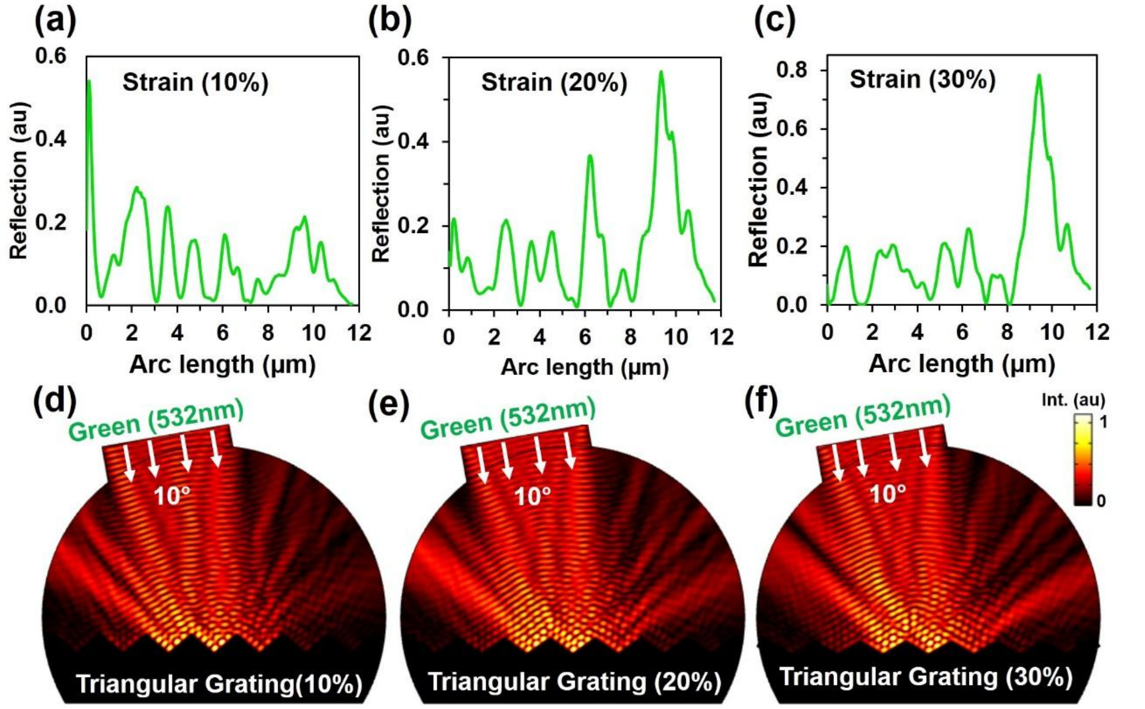

Figure S8: Computation modeling of retoreflection, diffraction/scattering from triangular grating structure with strain variation (10, 20, 30%) and green light 10° illumination.

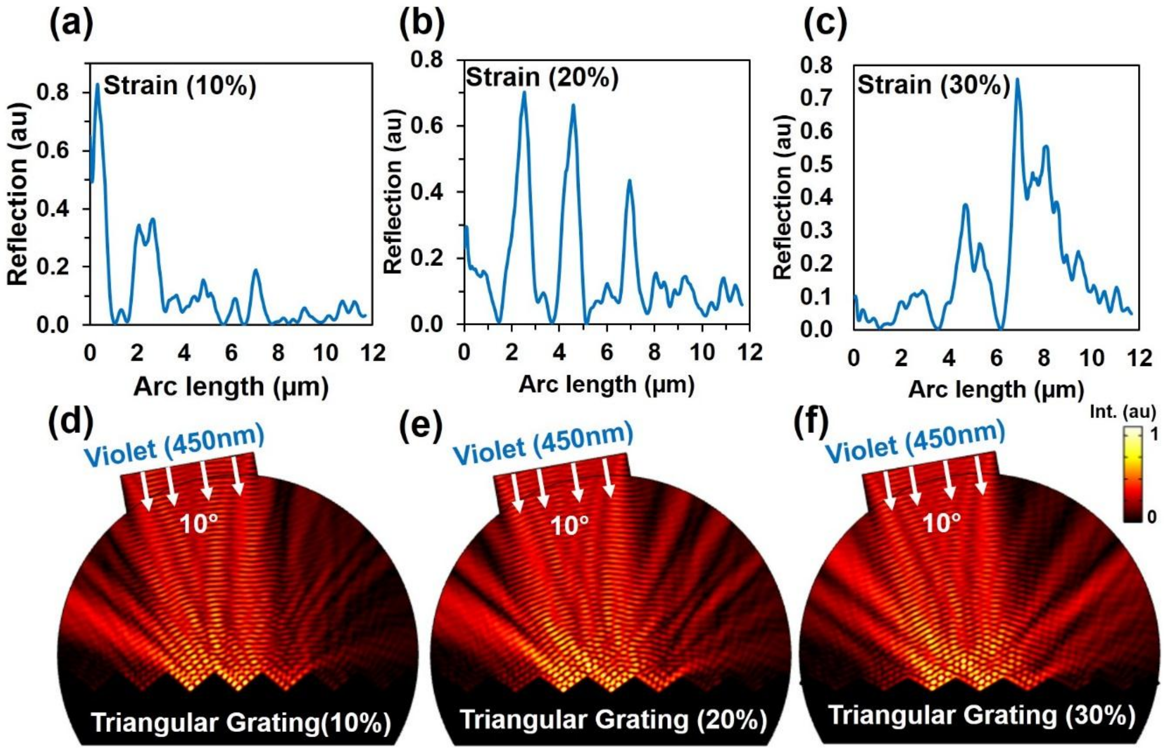

Figure S9: Computation modeling of retoreflection, diffraction/scattering from triangular grating structure with strain variation (10, 20, 30%) and blue light 10° illumination.

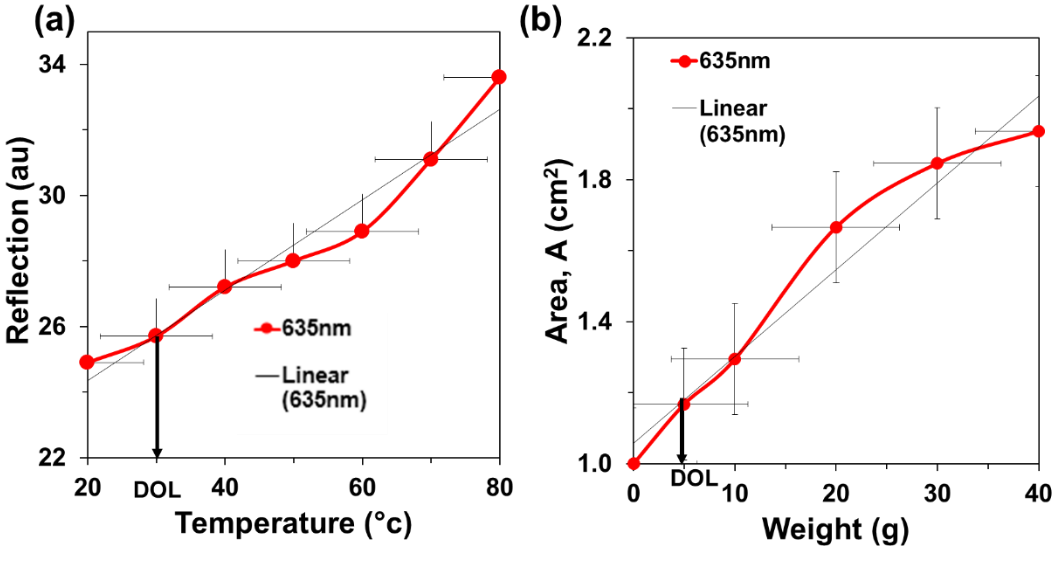

Figure S10: The detection limit (DOL) calculation for (a) temperature and (b) weight variation during red (635 nm) illumination.
